# Referral to Chinese medicine practitioners in Australian primary care: a survey of New South Wales rural and regional general practitioners

**DOI:** 10.1186/1749-8546-8-8

**Published:** 2013-04-08

**Authors:** Jonathan L Wardle, David W Sibbritt, Jon Adams

**Affiliations:** 1 Australian Research Centre in Complementary and Integrative Medicine, Faculty of Health, University of Technology Sydney, 235-253 Jones St, Ultimo, NSW, Australia; 2 Network of Researchers in the Public Health of Complementary and Alternative Medicine (NORPHCAM

## Abstract

**Background:**

Chinese medicine practitioners (CMPs) play an important part in rural and regional Australian healthcare. A survey was conducted to investigate referral practices between Chinese medicine (CM) and conventional primary health care practitioners in this region.

**Methods:**

A 27-item questionnaire was sent to all 1486 general practitioners (GPs) currently practising in rural and regional Divisions of General Practice in New South Wales, Australia. This survey explored GP opinions, perceptions and practices in relation to complementary and alternative medicine or Chinese medicine specifically.

**Results:**

A total of 585 GPs completed the questionnaire. Forty-nine were returned as ‘no longer at this address’, resulting in an adjusted response rate of 40.7%. One in ten GPs (9.9%) had referred their patients to CMPs at least a few times over the past 12 months, one in five GPs (17.4%) could not locate a CMP to refer to in their local area, and over one-third of GPs (37.7%) stated they would not refer to a CMP under any circumstances. GPs that had graduated from an Australian medical college (OR = 3.71; CI: 1.22, 11.23), GPs observing positive responses previously in patients using CM (OR = 2.53; 95% CI: 1.12, 8.58), GPs perceiving a lack of other options for patients (OR = 3.10; 95% CI: 1.12, 8.58), GPs reporting satisfactory or higher levels of CM knowledge (OR = 15.62; 95% CI: 5.47, 44.56), and GPs interested in increasing their complementary and alternative medicine knowledge (OR = 3.28; 95% CI: 1.17, 9.21) referred to CMPs more frequently than did other groups of GPs amongst the rural GPs included in this study.

**Conclusion:**

There has been little interaction between CMPs and Australian rural and regional GPs.

## Background

The use of complementary and alternative medicine (CAM) has emerged as an important public health issue in Australia [1,2], with patient visits to CAM practitioners accounting for half of all health consultations, and CAM accounting for half of all out-of-pocket healthcare costs [3]. Australian Chinese medicine practitioners (CMPs) provide approximately 15 million consultations annually, with 7.4% of Australians visiting a practitioner for acupuncture, 3.2% for Chinese manipulative therapy (*Tui Na*), 2.9% for *Tai Chi* or *Qi Gong* therapy, 2.3% for Chinese herbal medicine and 0.6% for Chinese dietetic therapy [3].

In Australia, acupuncture services are not necessarily provided by Chinese medicine (CM) practitioners practising CM principles [4,5]. However, CM does have a long history in Australia compared to other Western countries. The arrival of Chinese immigrants during the Victorian gold rushes helped establish the CM system, which was used extensively beyond the Chinese immigrant community by 1911 [6]. At the height of herbal medicine in Australia in the 1920s, one-fifth of all herbalists were practising CM [7]. Acupuncture was popularised by chiropractors and naturopaths in the late 1960s, and later by conventional medical professionals [4,8]. However, despite the long history and popularity of CM in Australia, few studies have investigated the relationship between CAM and the Australian health system [9-12].

The Australian Bureau of Statistics ranked CMPs the fifth largest groups of CAM primary care professions in Australia, after naturopaths, chiropractors, acupuncturists and osteopaths [13]. The Chinese Medicine Board of Australia allows traditional practitioners to register as comprehensive CMPs or practice-specific practitioners (such as acupuncturists or herbalists). Among traditionally trained practitioners, comprehensive CMPs form a larger professional group than do acupuncturists: 59.5% of registrants were qualified as comprehensive CMPs, with 37.9% qualified as acupuncturists only, 1.9% solely as Chinese herbalists, and the remainder as Chinese herbal dispensers [14]. The CM profession in Australia is growing significantly, with over one-quarter (27.3%) of practitioners entering the profession within the last 5 years [15]. The CM profession was recently included in the Australian national registration scheme for health practitioners, with most CMP training now being offered in the public university sector [16].

A few Australian studies have indicated significant levels of referral to CAM practitioners by general practitioners (GPs) [9-11], suggesting the existence of working relationships between CAM practitioners and GPs. GPs tend to prefer to refer to biomedical practitioners for CAMs more than to non-medically trained CAM practitioners [17]. Integration of CAM is an evolving and often controversial practice within the primary health care community in Australia [17,18], and as such, there is uneven integration, with different CAM therapies attracting different levels of support from and integration by GPs [9] (*e.g.*, some GPs may support some types of CAM, such as chiropractic, more than others, such as homeopathy).

Few studies have been conducted to investigate medical practitioners’ attitudes and behaviours with respect to specifc referrals to CMPs. Although acupuncture as a form of CAM appears to have good acceptance by the Australian GP community [9,11], GPs’ attitudes to other elements of CM practice remain mixed. This may be because biomedical practitioners regard CM as being pseudoscientific and incompatible with conventional medical principles [19].

A national GP survey [9] of the use of Chinese herbal medicine and Western herbal medicine found lower levels of use in practice and referrals by Australian GPs for Chinese herbal medicine than for Western herbal medicine. However, the survey also found that GPs perceived Chinese herbal medicine as being more effective than Western herbal medicine. However, to date there has been no comprehensive or focused investigation of GP attitudes (*e.g.*, whether GPs accept CM more than they do Western CAM) and practices in working with CMPs.

Geographical differences in CAM consumption were noticeable both in Australia and other countries [20], with increased use by rural populations over their urban counterparts [21], although a longitudinal investigation found no significant difference in acupuncture use between rural and urban Australian populations [22]. The prevalence and use of other forms of CM in rural and regional Australia have yet to be determined. In one study, acupuncturists and CMPs represented only 11% of the ‘primary care capable’ CAM practitioner workforce in rural and regional New South Wales [23], which is a lower proportion than these practitioners represent in national data [13]. CMPs may be consulted for very specific conditions; an exploratory study showed that nearly half of patients (47.1%) attending a private CM practice in rural Victoria saw the CM practitioner to treat pain, with a further 20.5% attending for fertility treatment and pregnancy management [24]. The results of a previous national workforce investigation suggested that treatment of musculoskeletal conditions is the primary reason Australians visit a CM practitioner [25].

This study aimed to investigate the patterns of referrals by GPs to CMPs in rural and regional New South Wales, Australia to fill this gap in the knowledge.

## Methods

A 27-item survey questionnaire (Additional file 1) was sent to all GPs practising in rural and regional General Practice Divisions of NSW, with a reminder card sent after two months. GPs were asked for their demographic and practice information and about their knowledge, attitudes, and practice and referral patterns to CMPs. CMPs were defined as traditionally trained practitioners who employ a variety of treatments (including acupuncture, physical therapy and herbal medicine) as required in accordance with diagnosis based on CM theory and principles. GPs were separately asked about referrals to acupuncture and herbal medicine practitioners. This distinction allowed for specific analysis of GP referral to practitioners who employ the ‘whole medical system’ of CM, rather than those who simply employ one practice traditionally associated with CM, but who do not necessarily practise within the Chinese medical model (for example, ‘medical’ acupuncture). Ethical approval for the study was obtained from the Ethics Committee of the School of Population Health Research of the University of Queensland (JW130508) and the Human Research Ethics Committee of the University of Newcastle (H-2008-0344).

Rural and regional areas were defined by their Rural, Remote and Metropolitan Area (RRMA) classifications [24]. The RRMA classification categorises areas based on population and remoteness as large or small metropolitan (1–2), large, small and other rural centres (3–5); and remote or other remote (6–7) (thus, a higher RRMA number reflects increasing rurality). All GPs practising in areas classified as RRMA 3–7 were invited to participate in this study. To minimise the effects of local variation, every rural and regional GP in Australia’s largest state (New South Wales) was surveyed. Rural and regional NSW was also specifically chosen as the study area because of research indicating a high prevalence of CM practitioners across this region, relative to other areas of Australia [23].

Survey questionnaire data were analysed by descriptive statistics *via* frequency distributions and cross-tabulations. Demographic and practice characteristics of GPs who referred patients to CMPs (at least a few times per year) and those who did so seldom or never were compared using chi-square tests. Multiple logistic regression modelling including all practitioner and practice characteristic variables was conducted by a backwards stepwise method of elimination using a likelihood ratio test to parsimoniously predict referral to CMPs. The threshold for statistical significance was set at α= 0.05. Data were analysed as described above using STATA 11 software (Stata Corporation, USA).

## Results

Questionnaires were sent to 1486 GPs practising in rural and regional GP divisions of NSW. A total of 585 completed questionnaires were returned. Forty-nine questionnaires were returned as ‘no longer at this address’, and the adjusted overall response rate was 40.7%. The respondents were aged 45–54 years and 53.5% of them were male. Over three-quarters of respondents (77.8%, n = 456) had completed their medical training at an Australian university, whilst less than 1% (0.9%, n = 5) had completed their medical training at a university in China. Aside from a slight (46.5% in this study *vs.* 39% nationally) over-representation of women, the respondent profile was broadly representative of the GP community in the study area [26].

Rates of referral from rural GPs to CMPs are shown in Table 1. Only a small number of GPs (2.7%, n = 16) referred to a CMP at least once per month, although nearly one in ten (9.9%, n = 58) GPs referred at least a few times per year. Over one-third (37.7%, n = 221) of GPs stated that they would never refer to a CMP under any circumstances. Nearly one-fifth (17.4%, n = 102) of GPs were unaware of local CMPs in their local area, and were therefore unable to refer. A very small number of GPs (1.4%; n = 8) had a personal professional relationship with a specific individual CMP. No GPs in this study reported practising CM.

**Table 1:** Rates of referral by rural GPs to CMPs in the past 12 months

**Referral rate**	**Frequency (%)**
At least weekly	7 (1.2%)
At least monthly	9 (1.5%)
A few times per year	42 (7.2%)
I have not referred but would consider	201 (34.3%)
I would never refer	221 (37.7%)
I have not referred as I do not know of any practitioners	102 (17.4%)
Total have never referred	524 (89.6%)
No response	3 (0.5%)

Table 2 shows a comparison between GPs who referred to a CMP at least a few times per year and those who did not by demographic characteristics. Level of rurality (RRMA) was significantly associated (*P* = 0.016) with higher referral to CM, although no consistent pattern was apparent. None of the other demographic factors analysed was significantly associated with referral to CMPs.

**Table 2:** Demographic and practice characteristics associated with referral to CMPs by rural and regional GPs in New South Wales, Australia

		**Referral to CM practitioners**		
**Demographic characteristics**		**Weekly or monthly**	**Seldom or never**	***P *value**
		(%)	(%)	
Sex	Male	45.9	54.4	0.210
	Female	54.1	45.6	
Age	25-34	4.9	9.4	0.373
	35-44	24.6	21.2	
	45-54	32.8	38.4	
	55-64	32.8	23.9	
	> 65	4.9	7.3	
RRMA (Level of rurality)	3 – Least remote	31.2	28.1	0.016
	4	41.0	41.8	
	5	14.8	26.3	
	6	9.8	1.9	
	7 – Most remote	3.2	1.9	
Australian graduate?	Yes	78.7	77.9	0.883
	No	21.3	22.1	
Initially from a rural area?	Yes	21.3	33.6	0.055
	No	78.7	66.4	
Patient load (per week)	< 50	23.0	15.3	0.369
	51-100	39.3	35.7	
	101-150	23.0	30.5	
	151-200	14.8	12.4	
	> 200	0.0	6.1	

Table 3 shows a comparison between GPs who referred to a CMP at least a few times per year and those who did not by other factors. Referral to a CMP was significantly associated with increasing GP knowledge of CM (*P* < 0.001), the number of patients asking the GP about CAM (*P* < 0.001), personal CAM use by the GP for their own health (*P* < 0.001), patient requests for referral (*P* = 0.021), the GP perceiving that the patient does not have other options available (*P* < 0.001), the GP having experienced positive results with CM previously (*P* < 0.001), the GP using CAM practitioners as a major source of CAM information (*P* = 0.017), the GP’s belief in CM efficacy (*P* < 0.001), the GP being interested in increasing CAM knowledge (*P* < 0.001), and the GP being comfortable with referral to a CMP (*P* < 0.001).

**Table 3:** Other factors associated with referral to CMPs by rural and regional GPs in New South Wales, Australia

		**Referral to CM practitioner**		
**Factors**		**Weekly or monthly**	**Seldom or never**	***P *value**
		(%)	(%)	
Level of knowledge on CM	Excellent	1.3	9.8	< 0.001
	Very Good	4.2	31.2	
	Satisfactory	20.0	41.0	
	Poor	51.2	8.2	
	Very Poor	23.2	9.8	
Percentage of patients who have asked about CAM	< 10%	36.1	35.3	< 0.001
	11-25%	8.2	46.4	
	26-50%	6.6	8.4	
	> 50%	49.2	9.9	
Personal CAM use by GP	Regularly	54.1	8.0	< 0.001
	Often	21.3	17.4	
	Once/Rarely	14.8	32.6	
	Never, but would consider	9.8	13.4	
	Never, and would not consider	0.0	27.3	
Access to medical specialists in areas is a problem	Yes	4.9	2.5	0.279
	No	95.1	97.5	
Patient request for referral to CM	Yes	29.5	45.2	0.021
	No	70.5	54.8	
Lack of other options available for treatment	Yes	19.7	10.3	< 0.001
	No	75.4	89.7	
Positive results with CM previously	Yes	83.6	44.5	< 0.001
	No	16.4	55.5	
CAM practitioner are a major source of CAM information	Yes	31.2	18.1	0.017
	No	68.8	81.9	
Patients are a major source of CAM information	Yes	41.0	47.9	0.307
	No	59.0	52.1	
Belief in efficacy of CM?	Yes	90.2	37.8	< 0.001
	No	9.8	62.2	
GP Interested in increasing CAM knowledge?	Yes	83.6	51.9	< 0.001
	No	16.4	48.1	
Have prescribed CAM to patients previously?	Yes	78.7	71.2	0.219
	No	21.3	28.8	
GP comfort level with CM	Comfortable in general	51.7	3.5	< 0.001
	Only in specific circumstances	31.0	12.4	
	Only if I knew them in person	17.2	26.5	
	I would not refer	0.0	57.1	

### Predictive factors

The results of multiple logistic regression modelling to determine independent predictive factors for referring to CMPs are shown in Table 4. Increased knowledge of CM was associated with higher referral rates by GPs, with GPs reporting good or very good knowledge of TCM being 135.23 (95% CI: 34.89, 524.17) times more likely to refer to CM at least a few times per year than GPs who reported poor or very poor knowledge of CM. This odds ratio is unusually high, and is likely to have been inflated by the small sample size. GPs who graduated from Australian medical colleges were 3.71 (95% CI: 1.22, 11.23) time more likely to refer to CM at least once per month than were international medical graduates. GPs who had seen positive results from CM previously were 2.53 (95% CI: 1.12, 8.58) times more likely to refer to CM at least once per month than were those who had not. GPs were 3.10 (95% CI: 1.12, 8.58) more likely to refer to CMPs at least once per month if they perceived there were no other options available for patients than were GPs who did not. GPs who were interested in learning more about CAM were 3.28 (95% CI: 1.17, 9.21) times more likely to refer to CM than were those who were not.

**Table 4:** Predictive factors for referral by GPs to CMPs at least a few times per year by rural and regional GPs in New South Wales, Australia

**Factors**		**Odds ratio**	**95% CI**
Level of knowledge of CM	Poor or very poor	1.00	**—**
	Satisfactory	15.62	5.47, 44.56
	Excellent or good	135.23	34.89, 524.17
Lack of other options available for treatment	No	1.00	**—**
	Yes	3.10	1.12, 8.58
Positive results with CM previously	No	1.00	**—**
	Yes	2.53	1.72, 13.29
Interested in increasing CAM knowledge	No	1.00	**—**
	Yes	3.28	1.17, 9.21
Australian graduate?	No	1.00	**—**
	Yes	3.71	1.22, 11.23

## Discussion

To our knowledge, this is the first focused examination of GP referral to CMPs in rural and regional primary care in Australia. One in ten GPs (9.9%, n = 58) in this study had referred patients to a CMP in the past year. Only a small number of GPs had formal professional relationships with CMPs in this study. The low level of referral and professional relationships in this study could be related to the level of access to CMPs in rural and regional Australia, as the number of CMPs as a proportion of total CAM practitioners appears to be lower in rural and regional areas of Australia than it is in urban areas [23]. The results of our study indicated that 17.4% of GPs were completely unaware of any CMP in their local area. Exploration of the impact of practitioner presence and distribution upon CAM use and the referral practices of conventional healthcare providers to CAM practitioners would offer valuable insights to help inform policy and practice in relation to these growing and emerging professions in rural and regional areas.

However, low levels of referral and integration could also reflect deliberate avoidance of CM by conventional practitioners. A significant number of the GPs in this study exhibited strong negative attitudes towards referral to CMPs, with over one-third (37.7%, n = 221) reporting that they would not refer to a CMP under any circumstances. The validity of CM theory has recently been questioned in high-profile Australian biomedical journals, labelling CM as wholly unscientific and discouraging further research or practice in the discipline [19]. However, this finding directly contrasts with the high levels of support for individual elements of CM practice amongst Australian GPs. For example, the low rates of referral and high levels of opposition to CM referral by GPs in this study appear to be at odds with the high rates of referral and high levels of support uncovered in previous national surveys of Australian GPs [9,11]. They would also appear to contrast with studies showing a high uptake of acupuncture by Australian GPs using acupuncture in practice, which ranges between 15.1 and 18.0% [4,9,12,27]. However, the use of individual therapies such as acupuncture by Australian GPs may not incorporate CM philosophy, and may only be supported when ‘medical’ forms are used [28]. This hypothesis is supported by findings from a previous study, which suggested that although high support for acupuncture referral existed amongst Australian GPs, high levels of support were only apparent when the acupuncture was performed by a medical practitioner, with lower levels of support for CAM providers performing these practices [10]. However, although CMPs in Australia are now recognised *via* inclusion in the National Registration Scheme, there has been little formal exploration of GPs’ attitudes towards CM as a whole medical system, as opposed to individual practices. Further investigations into what aspects of CM practice GPs in Australia find valuable could offer insights into whether traditional CM practices are being supported by GPs, or whether individual CM therapies are being ‘medicalised’ for use in general practice with little traditional philosophy being incorporated and retained.

The results of our study indicated that GPs graduating from an Australian institution were nearly four times as likely to refer to a CMP as GPs who were trained overseas. This could indicate cultural differences in the perceptions of CAM due partly to the long history and established nature of Chinese medicine in Australia, which may influence and inform Australian-trained GPs in their decisions related to CMPs. The number of GPs graduating from Chinese universities (n = 5; 0.9%) was too low to conduct a separate analysis. Investigations of the specific impacts of medical training location or cultural background on attitudes, perceptions and practices of CAM should be undertaken to explore the reasons underling these differences and the impact on primary healthcare in diverse and cross-cultural settings.

Regulatory mechanisms have enforced higher standards for Australian CMPs. The Australian state of Victoria was the first Western jurisdiction to regulate CMPs [29], and CMP training entered the Australian public university sector in the early 1990s [8]. Such regulatory and standards protections could assuage some potential fears about referral to CAM providers amongst GPs who were trained in the Australian healthcare setting, whilst these protections may not exist in other countries [9,11]. Exploration of the influence regulatory mechanisms and protections have on GP referral rates to CAM could ascertain the role of the policy landscape on CAM practice in primary care.

Rural and regional issues associated with patient CAM use and practice may not affect levels of referral to CMPs by GPs in this study, which contrasts with data indicating higher use of CAM by rural populations than by urban populations [21]. Higher CAM use in rural and regional areas may be related to lower levels of conventional healthcare providers (*e.g.*, specialists and allied health), which would align with the finding that lack of treatment options independently predicted referral to a CMP [30]. However, it was unknown whether this was influenced by the rural setting of this study. Although a lack of other treatment options for patients was predictive of increased referral rates to CMPs by the rural and regional Australian GPs in our study, it was accompanied by factors such as previous positive experiences, interest in CAM and increased CAM knowledge [9]. Further investigation of referral patterns to CMPs in the broader GP population, or comparative work with urban GPs, could help ascertain the roles played by geographic factors in the interactions between CAM and general practice.

Although limited to one state (New South Wales), the large and varied study area was chosen to be broadly representative of Australian rural and regional general practice demographics [26]. Nevertheless, the demographics of the GPs in this study compared with national statistics (drawn from rural and regional areas and exhibiting a higher proportion of females) should be considered in generalising the study’s results to the broader Australian general practice population.

Our study found that CM was not highly used in rural and regional general practice in Australia. Support for incorporation of CMPs into healthcare delivery by GPs was mixed: there was significant opposition to integrating CMPs into rural and regional primary health care by GPs in these areas, which contrasted with the high support for individual therapies such as acupuncture shown by previous studies. The use of self-reported data and possible recall bias inherent in retrospective collection of data over a 12 month period, and self-selection, may also have resulted in some form of response bias in this study.

Inclusion of CM in the national registration scheme and growth in practitioner numbers may increase the role CMPs play in rural and regional healthcare in Australia. When viewed in the context of the findings from this study, such an increase should serve as an impetus for increased research into Chinese medicine practice, policy and regulation in these areas.

## Conclusions

There has been little interaction between CMPs and Australian rural and regional GPs.

## Competing interests

The authors declare that they have no competing interests.

## Authors’ contributions

JLW, DS and JA designed the study. JLW, DS and JA performed the data analysis. JLW, DS and JA wrote the manuscript. All authors read and approved the final manuscript.

## Supplementary Material

Additional file 1 Rural General Practice Complementary and Alternative Medicine Survey. Click here for file
